# The Role of Putative Phosphatidylserine-Interactive Residues of Tissue Factor on Its Coagulant Activity at the Cell Surface

**DOI:** 10.1371/journal.pone.0158377

**Published:** 2016-06-27

**Authors:** Shabbir A. Ansari, Usha R. Pendurthi, Prosenjit Sen, L. Vijaya Mohan Rao

**Affiliations:** 1 Department of Cellular and Molecular Biology, The University of Texas Health Science Center at Tyler, Tyler, Texas, United Sates of America; 2 Department of Biological Chemistry, Indian Association for the Cultivation of Science, Kolkata, 700032, India; University of Pittsburgh School of Medicine, UNITED STATES

## Abstract

Exposure of phosphatidylserine (PS) on the outer leaflet of the cell membrane is thought to play a critical role in tissue factor (TF) decryption. Recent molecular dynamics simulation studies suggested that the TF ectodomain may directly interact with PS. To investigate the potential role of TF direct interaction with the cell surface phospholipids on basal TF activity and the enhanced TF activity following the decryption, one or all of the putative PS-interactive residues in the TF ectodomain were mutated and tested for their coagulant activity in cell systems. Out of the 9 selected TF mutants, five of them -TF_S160A_, TF_S161A_, TF_S162A_, TF_K165A_, and TF_D180A_- exhibited a similar TF coagulant activity to that of the wild-type TF. The specific activity of three mutants, TF_K159A_, TF_S163A,_ and TF_K166A_, was reduced substantially. Mutation of the glycine residue at the position 164 markedly abrogated the TF coagulant activity, resulting in ~90% inhibition. Mutation of all nine lipid binding residues together did not further decrease the activity of TF compared to TF_G164A_. A similar fold increase in TF activity was observed in wild-type TF and all TF mutants following the treatment of THP-1 cells with either calcium ionomycin or HgCl_2_, two agents that are commonly used to decrypt TF. Overall, our data show that a few select TF residues that are implicated in interacting with PS contribute to the TF coagulant activity at the cell surface. However, our data also indicate that TF regions outside of the putative lipid binding region may also contribute to PS-dependent decryption of TF.

## Introduction

Tissue factor (TF), a transmembrane glycoprotein, is the cofactor for the serine protease coagulation factor VIIa (FVIIa). The TF-FVIIa complex formed on the cell surface initiates the coagulation cascade via a limited proteolytic cleavage of clotting proteins, factor IX and factor X, ultimately leading to the formation of fibrin [1]. The formation of the TF-FVIIa complex not only initiates the coagulation process to maintain hemostasis but also transduces cell signaling through the cleavage of protease-activated receptors (PARs) [2,3]. Tissue factor is constitutively expressed on the surface of many extravascular cells, including fibroblasts and epithelial cells, but not in cells that come in contact with blood, such as monocytes and endothelial cells [4,5]. However, certain pathological conditions induce TF expression in monocytes and endothelial cells [6–8], which often leads to thrombotic disorders [9–11]. Thus, the precise regulation of TF expression and the activity on the surface of cells is not only essential to hemostasis but also health in general.

The majority of the TF present on the cell surface exists in the cryptic (inactive) state but transforms to the active state (decrypted) following cell activation or injury [12]. Mechanisms that regulate TF activity on the cell surface are not completely understood. Although various mechanisms have been proposed for TF decryption [13–17], exposure of phosphatidylserine (PS) on the outer cell surface membrane following cell perturbation is thought to be predominantly responsible for TF decryption [13,15,18,19]. Recent studies of molecular dynamics simulation of the TF ectodomain in solution and on the surface of anionic phospholipids suggested a direct interaction of PS head groups with specific residues in TF [20]. This interaction is thought to contribute to the optimal presentation of the TF exosite region to its protein substrates, factors IX and X, through modulation of conformation-specific changes in TF [20]. These data raise the possibility that a direct interaction between the lipids that have been exposed following cell perturbation and TF may play a role in TF decryption. The subsequent studies using purified TF relipidated in predefined PC/PS vesicles supported the significance of the interaction between a few of these specific amino acid residues in TF and PS for TF activity [21]. However, the involvement of these specific lipid binding residues of TF in TF decryption in a complex biological membrane is unknown.

In the present study, we investigated the potential role of TF direct interaction with the lipids on the cell surface through the putative lipid binding residues in supporting TF activity, both on unperturbed cells and cells stimulated to decrypt TF. For these studies, we generated a panel of plasmid and adenoviral constructs of TF variants and expressed them in two different cell model systems, and then determined their specific activity in unperturbed and perturbed conditions.

## Materials and Methods

### Reagents

Recombinant human FVIIa and affinity purified rabbit anti-human FVIIa polyclonal antibody were provided by the late Walter Kisiel, the University of New Mexico Health Science Center, Albuquerque, NM, USA. Purified human FX was purchased from Enzyme Research Laboratories (South Bend, IN, USA). Purified human FXa was obtained from Haematologic Technologies, Inc. (Essex Junction, VT, USA). Preparation and characterization of monospecific polyclonal antibodies against human TF was described previously [22]. TF10H10 and TF9C3 hybridomas were kindly provided by James H. Morrissey, University of Illinois, College of Medicine, Urbana, IL, USA. TF10H10 and TF9C3 mAbs were purified from mouse ascites fluid using the Affi-Gel Protein A MAPS II Kit from Bio-Rad (Hercules, CA, USA). FuGENE HD transfection reagent was from Promega (Madison, WI, USA). Hygromycin B was from A.G. Scientific (San Diego, CA, USA). HgCl_2_ and ionomycin were from Sigma-Aldrich (St. Louis, MO. USA). Alkaline phosphatase-labeled Streptavidin and BluePhos Microwell Phosphatase Substrate System were from KPL (Gaithersburg, MD, USA).

### Cell culture

CHO-K1 cells were obtained from the American Type Culture Collection (ATCC, Manassas, VA, USA) and cultured in F-12K medium supplemented with 10% fetal bovine serum (FBS) and 1% penicillin/streptomycin. Human monocytic leukemia cell line, THP-1, was obtained from ATCC and was cultured in RPMI 1640 medium supplemented with 10% FBS and 1% penicillin/streptomycin. Both the cell types were cultured at 37°C and 5% CO_2_ in a humidified incubator. The cells were washed once with buffer A (10 mM N-2-hydroxyethylpiperazine-N’-2-ethanesulfonic acid [HEPES], 0.15 M NaCl, 4 mM KCl, and 11 mM glucose, pH 7.5) prior to their use in experiments.

### Generation of TF mutant constructs by site-directed mutagenesis

Site-directed mutagenesis was performed using the QuikChange XL II Site-Directed Mutagenesis Kit (Stratagene) following the manufacturer’s protocol. The wild-type TF gene was cloned into the expression vector plasmid pcDNA3.1 hygro vector (Invitrogen) and pacAd5 CMVK-NpA shuttle vector (Cell Biolabs, San Diego, CA) and were used as the template for site-directed mutagenesis. To generate the different mutants of TF in the lipid binding region and to mutate all the putative lipid binding residues (LBR), the following synthetic oligonucleotides were designed:

TF-K159A(f) 5’ACACTTTATTATTGGGCATCTTCAAGTTCAGG3’

TF-K159A(r) 3’CCTGAACTTGAAGATGCCCAATAATAAAGTGT5’

TF-S160A(f) 5’CACTTTATTATTGGAAAGCTTCAAGTTCAGGAAAG3’

TF-S160A(r) 3’CTTTCCTGAACTTGAAGCTTTCCAATAATAAAGTG5’

TF-S162A(f) 5’ATTATTGGAAATCTTCAGCTTCAGGAAAGAAAACAG3’

TF-S162A(r) 3’CTGTTTTCTTTCCTGAAGCTGAAGATTTCCAATAAT5’

TF-S163A(f) 5’ATTGGAAATCTTCAAGTGCAGGAAAGAAAACAGCC3’,

TF-S163A(r) 3’GGCTGTTTTCTTTCCTGCACTTGAAGATTTCCAAT5’

TF-G164A(f) 5’GGAAATCTTCAAGTTCAGCAAAGAAAACAGCCAAAAC3’

TF-G164A(r) 3’GTTTTGGCTGTTTTCTTTGCTGAACTTGAAGATTTCC5’

TF-K165A(f) 5’AATCTTCAAGTTCAGGAGCGAAAACAGCCAAAACAA3’

TF-K165A(r) 3’TTGTTTTGGCTGTTTTCGCTCCTGAACTTGAAGATT5’

TF-K166A(f) 5’CTTCAAGTTCAGGAAAGGCAACAGCCAAAACAAACA3’

TF-K166A(r) 3’TGTTTGTTTTGGCTGTTGCCTTTCCTGAACTTGAAG5’

TF-D180A(f) 5’AGTTTTTGATTGATGTGGCTAAAGGAGAAAACTACTG3’

TF-D180A(r) 3’CAGTAGTTTTCTCCTTTAGCCACATCAATCAAAAAC5’

TF-K159-K166A(f) 5’ATACACTTTATTATTGGGCAGCTGCAGCTGCAGCAGCGGCAA3’

TF-K159-K166A(r) 3’TTGCC GCTGCTGCAGCTGCAGCTGCCCAATAATA AAGTGTAT5’.

TF-K159-K166A plasmid was used as a template to mutate the aspartate residue at position 180 to alanine to generate the TF_LBR_ mutant. All mutations were verified by DNA sequencing of the plasmids (DNA sequencing service was provided by Eurofins MWG/Operon, Huntsville, AL).

### Stable expression of TF mutants in CHO-K1 cells

For the generation of stable cell lines, CHO-K1 cells were seeded in 60 mm dishes, and the following day, they were transfected with one μg of plasmid DNA encoding either wild-type TF or one of the TF variants using FuGENE HD transfection reagent. The cells were grown in the presence of hygromycin B (800 μg/ml) containing medium to generate the stable cell lines. Stable cell lines were maintained under the selection pressure with hygromycin B.

### Generation of recombinant adenoviral constructs of wild-type TF and TF variants

TF pacAd5 CMVK-NpA shuttle vector and pacAd5 9.2–100 adenovirus backbone DNA were linearized with *PacI* for cotransfection into HEK293 AD cells. Cells were maintained in the incubator at 37°C, 5% CO_2_ for 12–14 days with an addition of 1 ml extra medium intermittently. The cells were harvested as described in the manufacturer’s protocol (Cell Biolabs) to obtain a high-titer virus. The viral titer was determined by the QuickTiter adenovirus immunoassay kit from Cell Biolabs.

### Adenoviral transduction of TF in THP-1 cells

THP-1 cells (2x10^5^) cultured in RPMI serum-rich medium were transduced with either wild-type TF or TF mutant adenoviruses (~100 moi/cell, the dose was adjusted slightly among various TF variants to obtain similar TF expression levels). The cells were grown for 48 h and washed twice with buffer A before they were used in experiments.

### Determination of ^125^I-FVIIa and ^125^I-TF mAb binding to cells

TF mAbs (9C3 and 10H10) and FVIIa were labeled with ^125^I using Iodo-Gen (Thermo Scientific, Rockford, IL, USA)-coated polypropylene tubes and Na^125^I (Perkin Elmer, Waltham, MA, USA) according to manufacturer’s protocol and as described previously [23,24]. A saturating concentration of the radiolabeled TF mAb or FVIIa (10 nM) were added to the cells in buffer B (buffer A containing 1 mg/ml bovine serum albumin [BSA], 5 mM CaCl_2_ and 1 mM MgCl_2_) and incubated for 2 h on ice in the cold room. To determine K_d_ for FVIIa binding to wild-type TF and TF mutants, cells were incubated with varying concentrations of ^125^I-FVIIa, 1 to 50 nM. At the end of 2 h incubation, the unbound radiolabeled ligand was removed from the cells. Cells were washed four times with ice-cold buffer B and the bound labeled protein was eluted by adding 100 mM glycine, pH 2.3 for 5 min. In the case of THP-1 cells, centrifugation was used (2,000 rpm for 4 min in an Eppendorf microfuge) to remove the unbound radioligand and the cells were washed 3 times with buffer B. The radioactivity from the glycine eluate or the total cell pellet was counted in a gamma counter. To determine non-specific binding, parallel binding studies were performed using control cells (cells not transfected with TF constructs), and these values were subtracted from the values obtained in cells transfected to express TF to determine the specific binding.

### TF activity assay

The procoagulant activity of the wild-type and mutant TF was measured in a FX activation assay as described previously [25]. Briefly, the cells were incubated with FVIIa (10 nM) for 5 min in buffer B at 37°C with gentle shaking. After 5 min incubation, FX (175nM) was added to the cells. FXa generation was allowed for 20 min for CHO-K1 cells and 4 min for THP-1 cells. During this phase, the rate of FX activation was linear, and only less than 10% of the substrate was activated. An aliquot removed from the reaction mixture was added to the stopping buffer (Tris-buffered saline containing 1 mg/ml BSA and 10 mM EDTA), and the amount of FXa generated was determined in a chromogenic assay with the use of the substrate Chromogenix S2765 as described earlier [25]. To decrypt TF, the cells were stimulated with 100 μM HgCl_2_ for 5 min or 10 μM ionomycin for 10 min just before the addition of FVIIa. A low basal endogenous TF activity of non-transfected THP-1 cells, which represents less than 10% of the activity observed in cells transduced with wild-type TF, was removed from TF activity measured in THP-1 cells transfected with wild-type TF and TF mutants to evaluate the specific activity of wild-type and TF mutants.

### Data collection and analysis

All experiments were done in duplicates at least 3 independent times. The data shown in the figures and tables represent mean ± SEM. When statistical significance was calculated, a t-test was used to determine whether TF activity observed in a TF mutant significantly differs from that of wild-type TF.

## Results

### Effect of specific mutations in the lipid binding region of TF on FX activation in CHO-K1 cells

**Fig 1:**
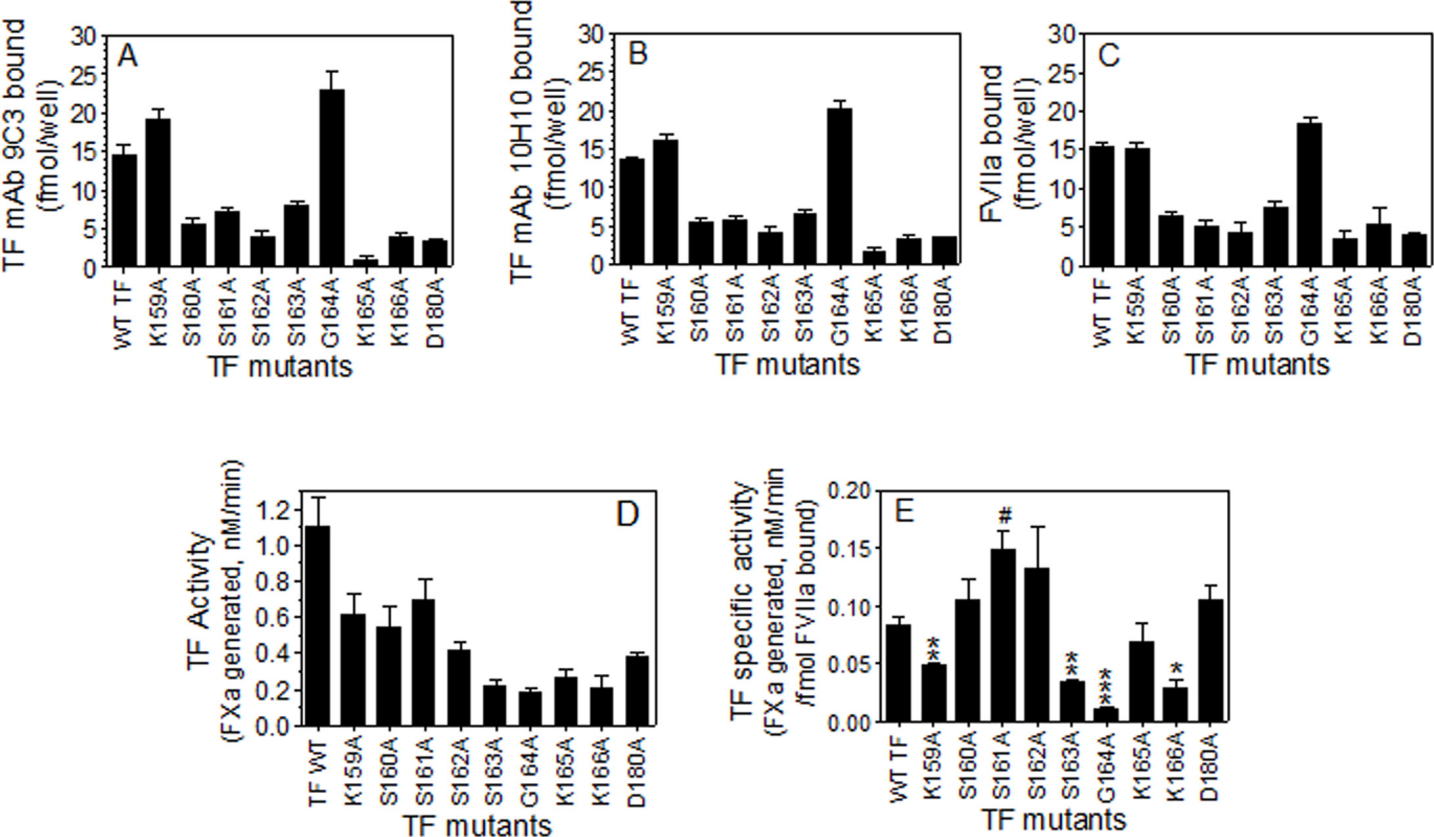
Analysis of tissue factor protein levels, FVIIa binding, and the coagulant activity of wild-type TF or lipid-interacting residue mutants of TF in CHO cells. CHO cells stably transfected to express wild-type TF or mutant TF, or untransfected CHO cells were incubated with ^125^I-labeled TF mAb 9C3 (A), 10H10 (B) or FVIIa (C) (10 nM) at 4°C for 2 h in buffer B. The concentration of the radioligand bound to cell surface TF was determined as described in the Methods. To determine the specific binding, the values obtained with untransfected cells were subtracted from the values obtained in cells expressing wild-type TF or mutant TF. In general, we observed 5 to 8% of TF mAb and 20 to 30% of FVIIa binding in untransfected cells compared to cells transfected with wild-type TF. (D) CHO cells expressing wild-type TF or a TF mutant were exposed to FVIIa (10 nM) and FX (175 nM) for 20 min, and the amount of FXa generated was determined in a chromogenic assay. Panel E depicts the TF activity as calculated by the amount of FXa generated (nM) per fmol TF-FVIIa complexes formed on the cell surface. The data shown in the figure represents the mean ± SEM from 3 independent experiments performed in duplicate. The specific activity of some of the mutants was statistically significantly lower than that of the wild-type TF (t-test). *, *P*< 0.05; **, *P*< 0.01; ***, *P*< 0.001. # indicates that specific activity of TF mutants was statistically significantly higher than that of the wild-type TF

Based on the recent molecular dynamic simulation studies [20] and studies performed with TF mutants in liposomes [21], we selected 9 different TF amino acid residues that were thought to be involved in TF interaction with anionic lipids to investigate the importance of TF ectodomain interaction with the cell surface membrane lipids on TF procoagulant activity in both basal state and following decryption. In initial studies, we used the CHO cell system to generate the cells expressing wild-type TF and TF mutant at the cell surface. Although CHO cells were transfected with equal amounts of plasmid DNA of wild-type TF and TF variants under identical experimental conditions, TF protein expression in the transfected cells was varied (data not shown). Therefore, to measure TF functional activity on the surface accurately in cells expressing wild-type TF and TF variants, we quantified TF expression levels at the cell surface by measuring the amount of ^125^I-labeled TF mAb bound to the cells. As shown in Fig 1A and 1B, two different TF mAb - 10H10 and 9C3—bound to the cells at nearly identical amounts. Next, we investigated FVIIa binding to TF mutants. Since all studies described here employed a FVIIa concentration that is equivalent to the plasma concentration of FVII (10 nM), FVIIa binding studies were carried out at this concentration. As shown in Fig 1C, the level of FVIIa bound to the cells expressing various TF mutants were essentially similar to that of the amount of TF mAb bound to these cells. These data indicate that all TF variants used in the study had no defect in their ability to interact with FVIIa, at least at the concentration of FVIIa (10 nM) used in the study. Thus, the differences in FVIIa binding levels observed in cells expressing the wild-type and variants reflect the differences in expression levels of TF at the cell surface in the transfected cells and not due to potential differences in FVIIa affinity to bind wild-type TF and TF mutants. Additional studies, in which we investigated FVIIa binding kinetics to wild-type TF and two select TF mutants (TF_S160A_ and TF_G164A_) using varying concentrations of FVIIa (1 to 50 nM), confirmed that FVIIa bound to TF mutants with a similar affinity as that of wild-type TF (K_d_, wild-type TF, 4.40±1.09 nM; TF_S160A_, 4.23+0.79 nM; TF_G164A_, 4.37±1.11 nM). This observation is consistent with the earlier finding where FVIIa binding to TF mutants was investigated in solution using an amidolytic assay [21]. Thus, the reduced TF activity observed with some of TF variants (Fig 1D) may reflect reduced expression of TF variants, compared to wild-type TF, at the cell surface. Therefore, TF activity was normalized to TF antigen levels and shown as TF specific activity (Fig 1E).

Out of the 9 selected TF mutants, five of them -TF_S160A_, TF_S161A_, TF_S162A_, TF_K165A_, and TF_D180A_- exhibited a similar or slightly higher TF coagulant activity to that of the wild-type TF. The differences in the TF-specific activity among them are not statistically significant except for one mutant (TF_S161A_) (Fig 1E). The specific activity of TF_K159A_, TF_S163A_ and TF_K166A_ mutants was reduced substantially, and in the range of 40% - 70% of wild-type TF. Mutation of the glycine residue at the position 164 markedly abrogated the TF coagulant activity, resulting in a 90% loss of TF-specific activity relative to the TF-specific activity of the wild-type (Fig 1E).

### The role of putative lipid interacting residues of TF on TF-FVIIa activation of FX in monocytic cells

**Fig 2:**
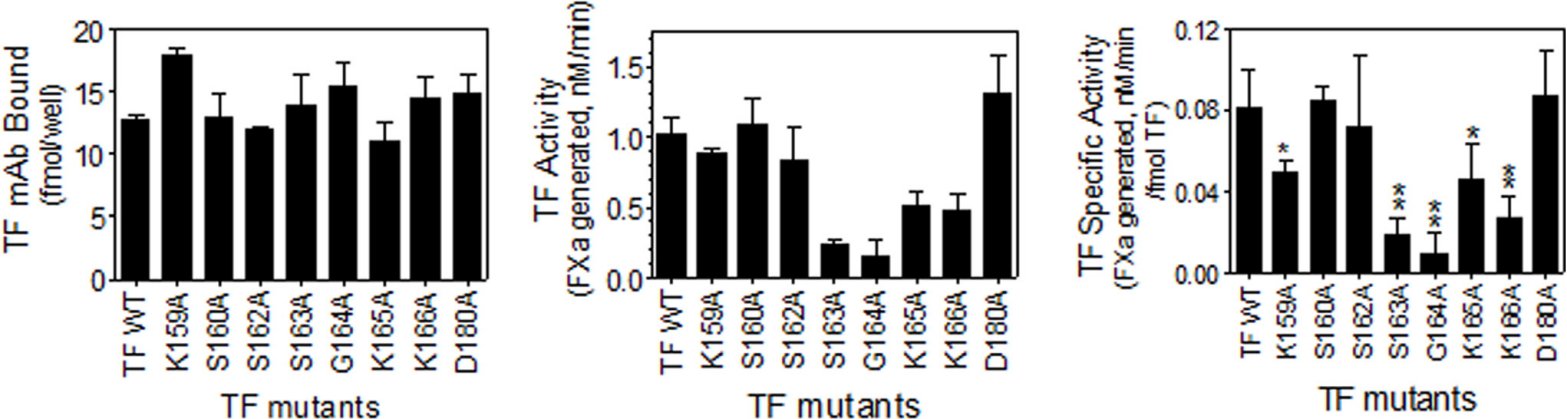
Determination of cell surface TF-specific activity in THP-1 cells transduced to express wild-type TF or TF mutants of lipid-interacting residues. THP-1 cells were transduced with adenovirus encoding wild-type TF or mutant TF. TF protein expression levels on the cell surface were determined by TF mAb 9C3 binding to the cells (A) and the cell surface TF activity was determined in FXa generation assay (B). TF-specific activity on the cell surface (C) was determined as the amount of FXa generated (nM)/fmol TF. The data shown in the figure represents the mean ± SEM from 3 independent experiments performed in duplicate. *, *P*< 0.05; **, *P*< 0.01 compared to the wild-type TF (t-test).

Although our earlier studies [26] indicated that TF coagulant activity in various cell types was regulated essentially in a similar manner, other studies in the literature suggest that TF encryption and decryption may vary among various cell types since the fold increase in TF activity following the cell stimulation was much higher in monocytic cells compared to other cell types [27]. Therefore, we next investigated the effect of select mutations in the putative lipid binding region of TF on TF functional activity in THP-1 monocytic cells. Since unperturbed THP-1 cells express very low levels of TF endogenously, it is feasible to transfect these cells with TF mutants to investigate the effect of TF mutations on its functional activity without much interference from the endogenous TF. As THP-1 cells were resistant to plasmid transfection, TF expression was transduced in these cells by adenoviruses encoding the wild-type TF or different mutants of TF. Measurement of TF expression levels showed a similar level of TF expression at the cell surface in cells transduced with adenoviruses encoding wild-type TF and TF variants (Fig 2A). Measurement of cell surface TF activity showed that some of the TF variants exhibited a similar level of TF activity seen with wild-type TF and other variants showed reduced activity (Fig 2B). As observed in the CHO cell system, TF_S160A_, TF_S162A_, and TF_D180A_ expressed TF-specific activity equivalent to that of the wild-type TF whereas TF_K159A_, TF_S163A_ and TF_K166A_ mutants exhibited significantly reduced cell surface TF activity compared to that of wild-type TF (40 to 75% inhibition) (Fig 2C). The mutation of G164 residue abolished the activity of TF by about 90%. Although minor differences exist in the specific activity of TF mutants in comparison to wild-type TF in CHO and THP-1 cell systems (Figs 1E and 2C, Table 1), overall the data are consistent in demonstrating that mutation of K159, S163, G164, and K166 residues significantly impaired TF-FVIIa activation of FX whereas mutation of S160, S162 and D180 residues had no significant effect.

**Table 1:** The ability of TF lipid binding mutants to support FX activation on the cell surface, and comparison of the present data to the data obtained in TF liposomes for the same mutants in an earlier study [21].

TF Mutant	CHO^a^	THP-1^a^	PC/PS vesicles^b^
**K159A**	49 ± 3.1	61 ± 4.3	23 ± 3.2
**S160A**	118 ± 4.2	105 ± 5.7	66 ± 3.6
**S161A**	141 ± 18	-	114
**S162A**	130 ± 6.2	89 ± 26.2	53 ± 1.3
**S163A**	38 ± 2.4	23 ± 6.1	5 ± 0.7
**G164A**	12 ± 0.9	11 ± 7.8	5 ± 0.5
**K165A**	94 ± 33.9	57 ± 8.9	14 ± 0.7
**K166A**	66 ± 4.4	34 ± 5.8	6.4 ± 0.5
**D180A**	133 ± 4.5	109 ± 15.9	64 ± 10

^a^Data shown was the normalized TF-specific activity, where TF-specific activity observed in cells expressing wild-type TF was taken as 100%. TF-specific activity as calculated by the amount of FXa generated (nM)/fmol TF present on the cell surface. In TF-transfected CHO cells, TF levels at the cell surface were determined in radioligand binding studies using saturating concentrations of two different TF mAb (10H10 and 9C3) and FVIIa as well as unlabeled FVIIa (levels of FVIIa bound to TF were determined in ELISA). The mean of all four determinants was taken as the number of TF molecules present on the cell surface to determine TF-specific activity. In THP-1 cells, TF levels at the cell surface were determined in radioligand binding studies employing 9C3 TF mAb. The data shown in the table represent mean ± SEM from 3 to 6 independent experiments.

^b^Data were mean ± SD of normalized rates of FX activation of TF mutants relipidated in 5%PS/95% PC vesicles, reported by Ke and Morrissey [21].

To address the concern whether the decreased FXa generation seen with the select TF mutants is due to an impaired substrate interaction with TF-FVIIa complex, we investigated Michaelis-Menten kinetics of FX activation by wild-type TF-FVIIa or mutant TF-FVIIa complexes. For these studies, we selected wild-type TF, two of the TF mutants (TF_S163A_ and TF_G164A_) that showed a significant decrease in FX activation and one of the TF mutants (TF_S160A_) that showed no apparent defect in supporting FX activation. Results of this study suggested that there were no significant statistical differences in Km values between wild-type TF and TF mutants (Table 2).

**Table 2:** Kinetic constants of TF-FVIIa mediated activation of FX in THP-1 cells transduced to express either wild-type TF or putative lipid binding TF mutants (n = 4; mean ±SEM).

TF Mutant	Kcat (min^-1)^	Km (nM)	Kcat/Km (M^-1^S^-1^)
**WT**	25.33 ± 0.616	134.40 ± 17.86	3.15 X 10^6^
**S160A**	26.86 ± 8.22	116.85 ± 31.07	3.84 X 10^6^
**S163A**	8.49 ± 0.098	139.20 ± 13.61	1.02 X 10^6^
**G164A**	7.68 ± 0.33	191.20 ± 12.80	6.71 X 10^5^

### The role of lipid-interacting residues of TF in TF decryption

**Fig 3:**
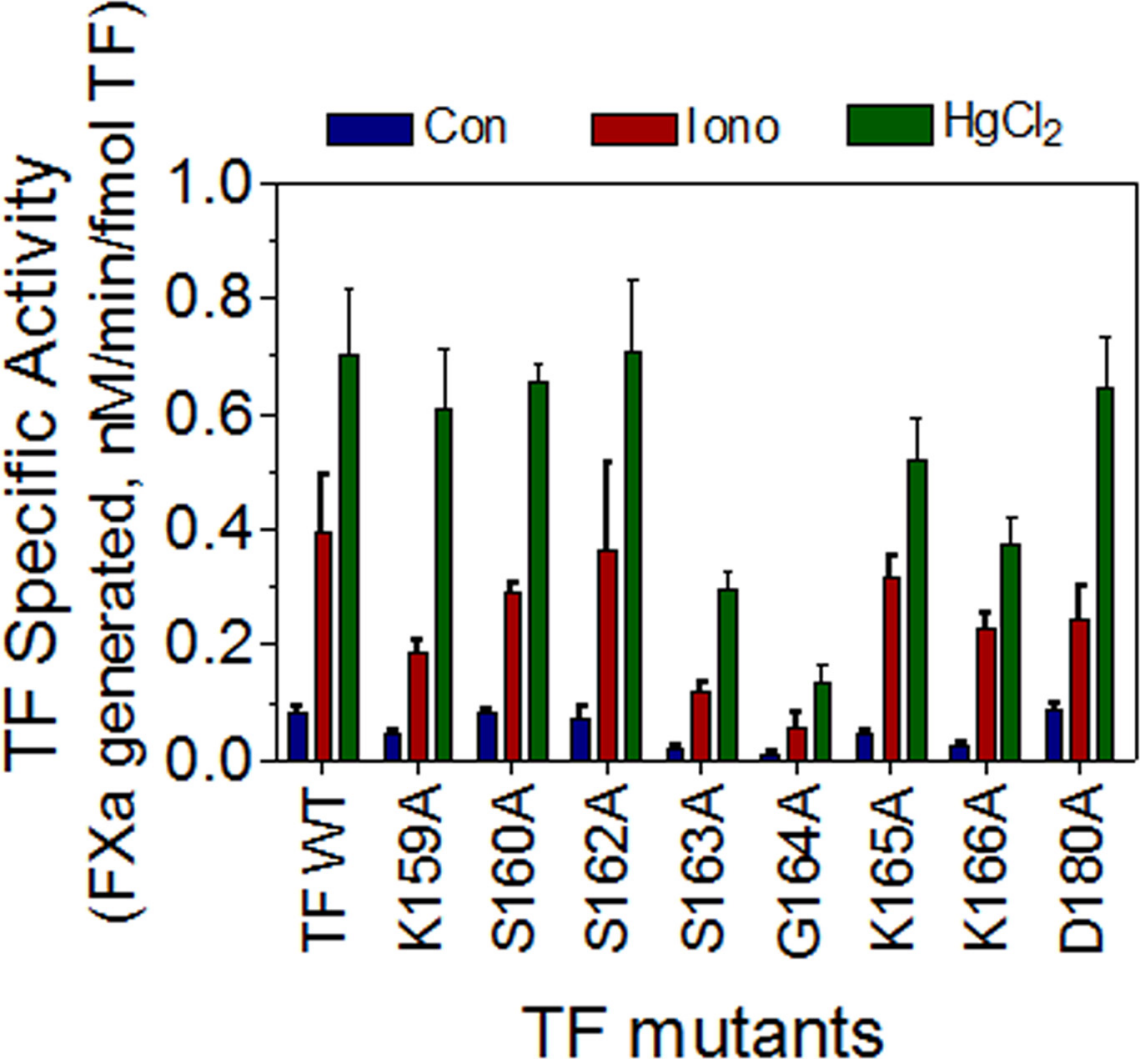
Effect of mutations in the lipid binding residues of TF on its decryption. THP-1 cells transduced to express wild-type TF or TF mutants (1 x10^6^ cells/ml) were treated with a control vehicle, calcium ionomycin (10 μM for 10 min) or HgCl_2_ (100 μM for 5 min). Then, FVIIa (10 nM) and FX (175 nM) were added to the cells, and the rate of FX activation was measured as described in the Methods. In a parallel experiment, THP-1 cells from the same batch that were used for FX activation studies were incubated with ^125^I-labeled TF mAb 9C3 for 2 h at 4°C to measure TF protein expression levels on the cell surface. The data obtained in these two assays were used to calculate the specific activity of the wild-type and mutant TF. The data shown in the figure represents the mean ± SEM from 3 to 6 independent experiments performed in duplicate.

THP-1 cells expressing wild-type or TF mutants in the putative lipid binding region were treated with calcium ionomycin (10 μM) or HgCl_2_ (100 μM) to decrypt TF. As shown in Fig 3 and Table 3, TF coagulant activity of THP-1 cells expressing the wild-type TF was increased by 4-6-fold upon ionomycin treatment, and 8 to 10-fold following HgCl_2_ treatment. A higher level of TF decryption in HgCl_2_-treated cells compared to ionomycin-treated cells may be due to differences in PS exposure at the cell surface or operation of an additional decryption mechanism in HgCl_2_-treated cells [28]. Calcium ionomycin and HgCl_2_ also increased the coagulant activity of TF mutants to a similar fold or higher than that observed in wild-type TF. The TF mutant, TF_G164A_, which exhibited a marked loss of TF activity, also responded robustly to ionomycin and HgCl_2_ treatments. Ionomycin and HgCl_2_ treatments increased the TF-specific activity of the mutant by about 6- and 15-fold, respectively. However, it is important to note that the TF-specific activity of the mutant was still substantially lower compared to the wild-type and other TF mutants. Overall, these data indicate that the mutation of selective residues in the lipid binding region fails to abrogate the PS-dependent TF decryption.

**Table 3:** Fold increase in TF coagulant activity of wild-type TF or lipid binding TF mutants following cell stimulation with calcium ionomycin or HgCl_2_.

TF Mutant	Ionomycin	HgCl_2_
**WT**	4.9 ± 1.20	8.7 ± 1.44
**K159A**	3.8 ± 0.42	12.4 ± 2.11
**S160A**	3.5 ± 0.17	7.8 ± 0.43
**S162A**	5.1 ± 2.12	10.0 ± 1.80
**S163A**	6.5 ± 0.73	16.3 ± 1.61
**G164A**	6.5 ± 2.77	15.2 ± 3.11
**K165A**	6.9 ± 0.85	11.5 ± 1.60
**K166A**	8.5 ± 0.95	13.8 ± 1.77
**D180A**	2.8 ± 0.61	7.4 ± 1.06
**LBR**	2.2 ± 0.88	11.1 ± 1.30

THP-1 cells expressing wild-type TF or mutant TF were treated with a control vehicle, calcium ionomycin or HgCl_2_ as described in Fig 3. Fold increase in TF coagulant activity was calculated relative to the TF-specific activity measured in the corresponding wild-type or TF mutant cells treated with a control vehicle (the fold increase shown in the table represents an average of data from 3 to 6 experiments). The fold-increase in TF coagulant activity in various TF mutants did not differ in statistically significant fashion when compared to the fold-increase observed in wild-type TF except in one case (S163 A mutant in HgCl_2_ treatment).

**Fig 4:**
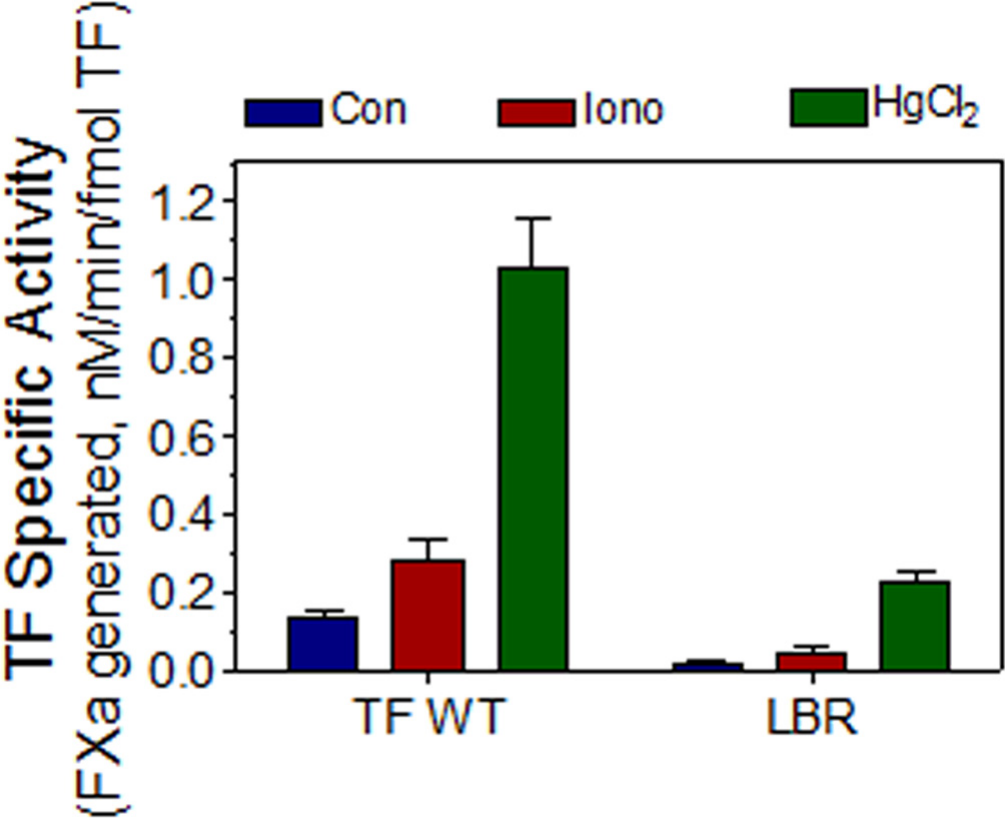
Mutation of multiple putative membrane binding residues of TF fails to abrogate the increased TF procoagulant activity following its decryption. To generate the lipid binding region mutant (LBR) of TF, all amino acid residues in the lipid binding region, i.e., K159 to K166 and D180, were mutated to alanine. The experiment was carried out essentially as described in the legend to Fig 3. Results were presented as means ± SEM of six independent experiments except the control, which was the mean of five experiments.

It is possible that interaction of not one, but multiple lipid binding residues with PS may be responsible for TF decryption following the PS exposure. To investigate this, we mutated all putative lipid binding residues in TF (TF_LBR_, LBR, lipid binding region) and used this construct to transduce TF expression in THP-1 cells. As observed with other TF mutants, TF_LBR_ mutant is capable of interacting with FVIIa with a similar affinity as that of wild-type TF (K_d_, wild-type TF, 4.4±1.1 nM; TF_LBR_, 3.9+0.85 nM). The specific activity of TF_LBR_ was markedly lower than that of the wild-type TF. Nonetheless, this mutant still exhibited about 10 to 15% of the activity of wild-type TF, similar to that of TF_G164A._ More importantly, TF activity of TF_LBR_ mutant also increased its activity by many fold in THP-1 cells following the stimulation with ionomycin or HgCl_2_ treatment (Fig 4), indicating that the mutant could undergo decryption.

**Fig 5:**
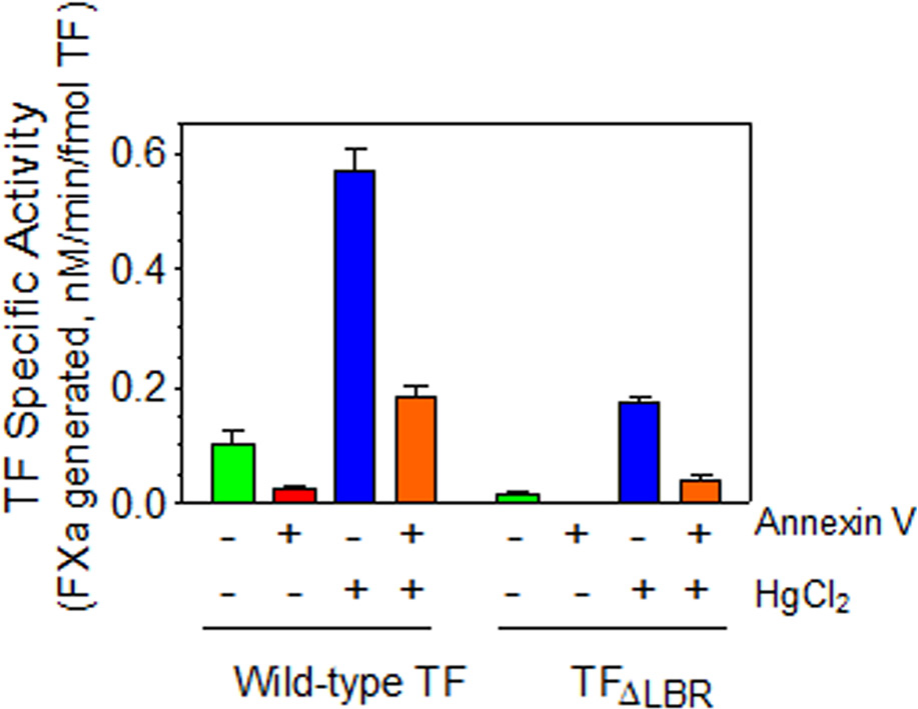
PS-dependent decryption of TF mutant lacking the PS-interactive residues. THP-1 cells expressing equal amounts of wild-type TF (TF WT) or TF mutant of the lipid binding region (TF_LBR_) were treated with a control vehicle or HgCl_2_ (100 μM for 5 min) in the presence or absence of annexin V (400 nM). TF-FVIIa activation of FX was measured by adding FVIIa (10 nM) and FX (175 nM) to the cells. In parallel wells, cells were incubated with ^125^I-labeled TF mAb 9C3 to measure TF protein expression levels on the cell surface. The data obtained in these two assays were used to calculate the specific activity of wild-type and mutant TF. The data shown in the figure represents the mean ± SEM from 3 independent experiments performed in duplicate.

**Fig 6:**
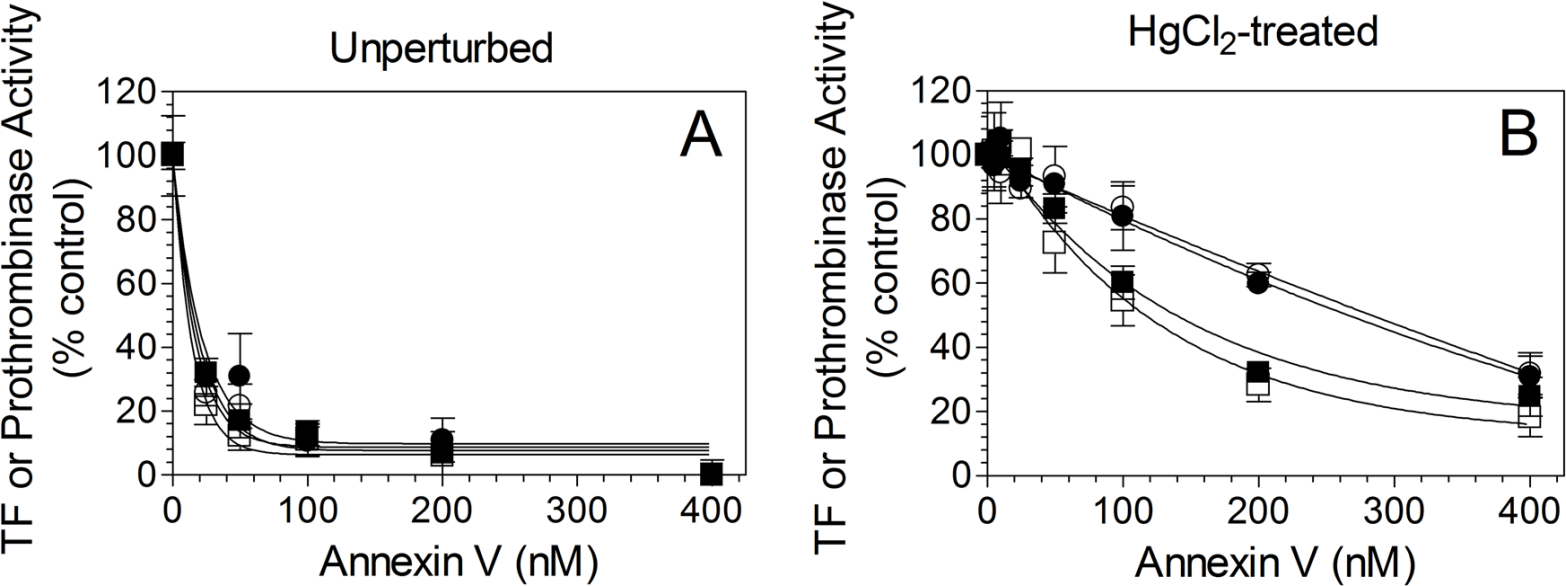
Annexin V inhibition of tissue factor and prothrombinase activities in unperturbed and HgCl_2_-treated cells. THP-1 cells were transduced to express equal amounts of wild-type TF or LBR mutant. The cells were incubated with varying concentrations of annexin V and treated with a control vehicle (A) or HgCl_2_, 100 μM for 5 min (B). TF-FVIIa activation of FX was measured by adding FVIIa (10 nM) and FX (175 nM) and measuring the amount of FXa generated in a chromogenic assay. Prothrombinase activity was measured by adding FVa (10 nM), FXa (0.1 nM), and prothrombin (1. 4 μM) and measuring thrombin generation in a chromogenic assay. As expected, HgCl_2_ treatment increased TF activity and prothrombinase activity by about 10-fold. To compare annexin V inhibition of TF activity and prothrombinase activity, the activity observed in the absence of annexin V for each set was taken as 100%. Squares (□,■) represent TF activity, and circles (○,●) represent prothrombinase activity. Open symbols (□,○) represent THP-1 cells transduced to express wild-type TF, and the closed symbols denote THP-1 cells transduced to express LBR mutant. The data shown in the graph represents mean ± SEM of three independent experiments.

Next to investigate whether the increased TF activity in cells expressing TF_LBR_ treated with HgCl_2_ was dependent on the increased PS or independent of PS, both the control and HgCl_2_-treated cells were incubated with annexin V to block the PS before adding FVIIa and FX. As shown in Fig 5, annexin V markedly diminished TF-FVIIa activation of FX in both the cell types, either expressing wild-type TF or TF_LBR_, under the basal condition as well in HgCl_2_-treated cells. In additional studies, we examined the concentration dependence of annexin V on inhibiting TF and prothrombinase activities of THP-1 cells expressing wild-type TF and TF_LBR_. These studies showed that annexin V inhibited both TF (Fig 6A) and prothrombinase (Fig 6B) activity of wild-type and TF_LBR_ in a similar concentration-dependent manner on unperturbed cells. A 25 nM of annexin V inhibited both the activities by about 75%, and the inhibition reached almost 100% at 400 nM of annexin V (Fig 6A). This reflects availability of a basal level of PS at the cell surface of unperturbed cells and its role in supporting TF and prothrombinase activities on unperturbed cells. In cells treated with HgCl_2_, a much higher concentration of annexin V was required to inhibit both the TF and prothrombinase activity (Fig 6B). This was expected since HgCl_2_ treatment was shown to increase PS exposure at the cell surface [25] and thus requires a higher concentration of annexin V to block the increased PS levels at the cell surface. Measurement of prothrombinase activity, as the indicator of PS levels on the cell surface, showed 8 to 10-fold increase in the prothrombinase activity in HgCl_2_-treated cells compared to unperturbed cells (thrombin generated U/ml/min, unperturbed cells 7.4 ± 0.75; HgCl2 treated cells, 65.3 ± 4.17; n = 3). The failure of annexin V to completely inhibit HgCl_2_-induced increase in TF activity and prothrombinase (Figs 5 and 6B) may reflect the inability of annexin V to access readily all PS sites on the membrane at the concentrations used or a fraction of HgCl_2_-enhanced activities may be PS-independent as suggested earlier by others [28]. In additional studies, we investigated the effect of annexin V on FVIIa binding to THP-1 cells expressing wild-type TF or TF_LBR._ As noted in our earlier studies in other cell model systems [25,29], annexin V had no significant effect on FVIIa binding to TF, either the wild-type TF or the TF_LBR_ mutant, on THP-1 cells (data not shown).

Next, we investigated whether the enhanced TF-FVIIa activation of FX following the PS-dependent TF decryption stemmed from the increased catalytic activity of TF-FVIIa complex and not from the enhanced affinity of the substrate to the enzyme complex due to an increased FX binding to PS exposed on the cell surface. For this, we measured the kinetic constants of TF-FVIIa activation of FX in unperturbed and HgCl_2_-treated THP-1 cells expressing either wild-type TF or TF_LBR_ mutant. As shown in Table 4, HgCl_2_ treatment increased the catalytic activity of both wild-type TF and TF_LBR_ mutant but had no significant effect on the Km values of TF-FVIIa activation of FX. Therefore, it is unlikely that a potential increase in FX binding to the PS exposed following HgCl_2_ treatment was responsible for TF decryption. Overall, these data indicate that TF_LBR_ mutant could undergo PS-dependent decryption following HgCl_2_ treatment. This suggests that the interaction of TF region other than the LBR with PS may be responsible for the enhanced TF activity following PS exposure.

**Table 4:** Kinetic constants of TF-FVIIa mediated activation of FX in unperturbed and HgCl_2_-treated THP-1 cells.

TF Mutant	Treatment	Kcat (min^-1^)	Km (nM)	Kcat/Km (M^-1^S^-1^)
**TF WT**	Control	18.8 ± 3.25	186.6 ± 27.1	1.68 x 10^6^
**TF WT**	*HgCl*_*2*_	232.8 ± 45.18	137.4 ± 6.8	2.82 x 10^7^
**TF**_**LBR**_	Control	1.1 ± 0.27	196.0 ± 39.2	9.02 x 10^4^
**TF**_**LBR**_	*HgCl*_*2*_	5.83 ± 0.47	222.6 ± 57.6	4.36 x 10^5^

THP-1 cells were transduced to express either wild-type TF (TF WT) or TF mutant of the lipid binding region (TF_LBR_). The cells were treated with a control vehicle or HgCl_2_ (100 μM) for 5 min and assayed for TF coagulant activity in the presence of FVIIa (10 nM) and varying concentrations of FX (25 to 1000 nM) (n = 6, mean ± SEM).

## Discussion

It is generally accepted that most of the TF molecules on the surface of a resting cell exist in an encrypted state with very little procoagulant activity and that this state of TF must undergo decryption to become fully active [12,13]. Although a number of distinctive mechanisms have been proposed for TF decryption, most of the evidence in the literature suggests that levels of anionic phospholipids, such as phosphatidylserine (PS), in the outer leaflet of the plasma membrane regulates the activity status of TF at the cell surface [12]. It is well established that PS markedly enhances the enzymatic activity of TF-FVIIa towards its substrates, factors IX and X [13,30–32], but the molecular mechanisms by which PS augments the enzymatic activity of TF-FVIIa is not entirely known. Although the binding of FVIIa and FX to PS via the Gla (γ-carboxyglutamic acid) domain and spatial stabilization of the FVIIa catalytic site subsequent to its binding to TF contribute to a marked increase in TF-FVIIa activation of FX [30,33], they do not fully explain the increased TF-FVIIa activation of FX following TF decryption [12]. We speculated earlier that direct TF interaction with anionic phospholipids on the cell surface may play a critical role in regulating TF activity [23,34].

Recently, Ohkubo et al. [20], based on molecular dynamics simulations of the TF ectodomain on the membrane surface, identified direct interactions between certain TF residues and PS head groups on the membrane. In subsequent studies, it was shown that mutation of some of the TF residues predicted to interact with PS, particularly mutations in or near the flexible loop from Lys 159 to Gly 164, decreased the activity of TF incorporated into PS/PC liposomes [21]. In the present study, we investigated the potential contribution of interaction of these putative membrane interactive residues of TF with the cell surface membrane lipids in supporting TF activity. Our studies revealed that mutation of a few select PS interactive residues in TF reduced the activity of TF on the cell surface. Mutation of K159, S163, and K166 reduced the activity of TF between 40 to 75% of the wild-type whereas G164 mutation decreased the activity by about 90%. Our present data show that the relative contribution of PS-interacting residues of TF in supporting TF activity at the cell surface may vary from that of their effect on TF activity in solution or liposomes. When TF mutants of PS-interacting residues were incorporated into 5% PS/95% PC liposomes, five of the mutants–K159A, S163A, G164A, K165A, and K166A –showed a marked impairment in their ability to support TF-FVIIa activation of FX (~5% for S163A, G164A, and K166A; 15 to 25% for K159A and K165A compared to wild-type TF) [21]. However, at the cell surface, the defect in K159A, K165A and K166A mutants in supporting FX activation was not as severe as that observed in liposomes. A number of reports in the literature showed that a single mutation, either of K165 or K166, resulted in an almost complete loss (~95%) of TF activity in the relipidated system [21,35–37]. The addition of PS was shown to only partially restore the activity of the single mutants [21,35]. In contrast, a single mutation of K165 or K166 in TF had only a modest effect on the activity of TF at the cell surface. Our present data of these mutants in the cell system were consistent with an earlier observation [35]. These data suggest that the interaction of one of the lysine residues with the cell surface membrane may be sufficient to induce necessary conformational change in TF-FVIIa exosite to recognize the substrate. It is possible that these lysine residues could interact with other negatively charged membrane components present on the cell surface and this interaction could induce necessary conformational changes in TF for its activity.

The marked defect in the activity of TF_G164A_ mutant at the cell surface probably reflects the importance of this residue in the substrate recognition independent of the phospholipid membrane surface since a similar severe impairment of the activity was observed in this mutant both in solution (phospholipid-free) and in the presence of phospholipids [21,38]. However, we did not find any significant differences in the *k*_*m*_ of TF_G164A_-FVIIa activation of FX vs. wild-type TF-FVIIa activation of FX. G164 residue is located in the surface-exposed loop of TF. Being the smallest amino acid with a minimal side chain, the glycine residue in the loop may allow the necessary flexibility to TF to have the optimal configuration of TF-FVIIa complex to activate the substrate effectively [38]. Furthermore, recent studies showed that G164 plays a critical role in Mg^2+^-dependent rate enhancement of TF-FVIIa activation of FX [39]. One or both of the above reasons could be responsible for the marked decrease in TF activity in G164A mutant.

Mutation of S162 and D180 residues in the putative lipid binding region of TF had no effect on the activity of TF at the cell surface. These mutations were shown to decrease TF activity by about 40 to 50% in solution or liposomes [21]. It is interesting to note that mutation of all eight residues in TF that were shown to interact with PS and contribute to the exosite binding of the substrate did not fully abrogate the TF-FVIIa activation of FX on the cell surface, indicating that the residues outside of the lipid binding region may also contribute to TF interaction with the membrane and the substrate recognition.

As expected, decryption of wild-type TF in monocytic cells upon exposure to calcium ionomycin or HgCl_2_ increased the cell surface TF activity by ~5 and 10-fold, respectively. Interestingly, decryption of TF mutants, either mutation of a single amino acid residue in the putative lipid binding region or mutation of all critical residues in the lipid binding region, also increased the TF activity to a similar fold. These data are somewhat surprising since one would expect that the blockage of the direct interaction of TF with the PS exposed on the cell surface by mutating the putative lipid binding residues would block the PS-dependent increased TF activity. These data suggest that either increased PS following HgCl_2_ treatment is not responsible for the increased TF activity or the presumed interaction of the PS-interacting residues in the TF ectodomain with the membrane PS is not essential for TF decryption. We can rule out the former possibility because inhibition of PS on the cell surface with annexin V blocked the enhanced TF activity of both wild-type TF and TF_LBR_, indicating the decryption of TF_LBR_ is dependent on PS. It is possible that other putative PS-interacting residues that are not examined in the present study or residues that are yet to be identified for their membrane interaction potential might have contributed for PS-dependent decryption of TF_LBR._

Although it is generally accepted that TF on cell surfaces must undergo decryption to express maximal coagulant activity, mechanisms that are responsible for TF decryption are not well understood. A majority of the evidence in the literature suggests that levels of anionic phospholipids, such as PS, in the outer leaflet of the plasma membrane play a critical role in regulating TF procoagulant activity at the cell surface [12]. However, other mechanisms—such as thiol-disulfide exchange pathways involving protein disulfide isomerase (PDI), the thioredoxin system, cholesterol content in the plasma membrane and post-translational modifications of TF—may also contribute to TF decryption [14,15,40]. Although our studies failed to support [25,41], it had been reported earlier that HgCl_2_-mediated decryption was largely independent of PS [28]. TF decryption induced by calcium ionophore was also shown to be partly independent of PS [42]. Since the majority of the increased TF activity following HgCl_2_ treatment was blocked by annexin V, the protein that specifically binds PS, it is unlikely that near normal decryption of TF mutants of the lipid binding region following HgCl_2_ or ionomycin treatment could be largely independent of PS.

Overall, our present data suggest that a few select amino acid residues in TF ectodomain that are implicated in interacting directly with PS contribute to the TF coagulant activity at the cell surface. However, the regulation of TF activity at the cell surface milieu may be different from that of PC/PS vesicles. The potential interaction of the putative PS-interacting residues of TF with cell surface membrane lipids appears to be less critical than with lipids in the liposomes in supporting TF activity. PS-dependent decryption of TF on the cell surface does not depend solely on the interaction between the putative PS interactive residues of TF with PS exposed on cells following cell activation. It is possible that a TF region other than recently identified LBR may also be responsible for enhancing the TF activity following the PS exposure. Further studies are required to understand fully the structure/function relation of TF at the cell surface.
